# RAID: Regression Analysis–Based Inductive DNA Microarray for Precise Read-Across

**DOI:** 10.3389/fphar.2022.879907

**Published:** 2022-07-22

**Authors:** Yuto Amano, Masayuki Yamane, Hiroshi Honda

**Affiliations:** R&D Safety Science Research, Kao Corporation, Tochigi, Japan

**Keywords:** oligonucleotide array, gene expression analysis, hepatotoxicity, alternative method, new approach methodology

## Abstract

Chemical structure-based read-across represents a promising method for chemical toxicity evaluation without the need for animal testing; however, a chemical structure is not necessarily related to toxicity. Therefore, *in vitro* studies were often used for read-across reliability refinement; however, their external validity has been hindered by the gap between *in vitro* and *in vivo* conditions. Thus, we developed a virtual DNA microarray, regression analysis–based inductive DNA microarray (RAID), which quantitatively predicts *in vivo* gene expression profiles based on the chemical structure and/or *in vitro* transcriptome data. For each gene, elastic-net models were constructed using chemical descriptors and *in vitro* transcriptome data to predict *in vivo* data from *in vitro* data (*in vitro* to *in vivo* extrapolation; IVIVE). In feature selection, useful genes for assessing the quantitative structure–activity relationship (QSAR) and IVIVE were identified. Predicted transcriptome data derived from the RAID system reflected the *in vivo* gene expression profiles of characteristic hepatotoxic substances. Moreover, gene ontology and pathway analysis indicated that nuclear receptor-mediated xenobiotic response and metabolic activation are related to these gene expressions. The identified IVIVE-related genes were associated with fatty acid, xenobiotic, and drug metabolisms, indicating that *in vitro* studies were effective in evaluating these key events. Furthermore, validation studies revealed that chemical substances associated with these key events could be detected as hepatotoxic biosimilar substances. These results indicated that the RAID system could represent an alternative screening test for a repeated-dose toxicity test and toxicogenomics analyses. Our technology provides a critical solution for IVIVE-based read-across by considering the mode of action and chemical structures.

## Introduction

Non-animal testing to assess the efficacy and safety of chemical substances is one of the key concepts in balancing animal welfare and efficient development. Since the marketing ban in the EU in March 2013 [(EC) No. 1223/2009] (EU, 2009) of cosmetic products and ingredients tested on animal models, safety assessment methodologies independent of animal testing have attracted much attention. Simultaneously, the utilization of non-animal high-throughput technology for optimizing drug discovery processes is becoming highly important in pharmaceuticals (Loiodice et al., 2017; Rognan, 2017; Amano et al., 2020).

Read-across, a process that estimates substance toxicity based on the concept that substances with similar chemical structures have similar biological activities, represents a promising approach and has already been conceptually accepted as a reliable safety risk assessment by some regulatory authorities (ECHA, 2017; European Commission, 2018). Likewise, quantitative structure–activity relationship (QSAR) has been widely used, and impurity characterization received regulatory acceptance (ICH M7). However, since subtle structural differences may elicit different biological responses, supporting the read-across robustness by using biological similarities has been considered important (Ball et al., 2016, 2020; Zhu et al., 2016). Registration, Evaluation, Authorization, and Restriction of Chemicals (REACH) mentions that the read-across performed by registrants often fails to comply with the legal requirements due to defects in the hypothesis and justification of toxicological prediction (ECHA, 2020).

There are two approaches to enhance the reliability of read-across: 1) employment of *in vitro* data relevant to specific toxicity. Methodologies to incorporate *in vitro* data within read-across (Ball et al., 2016, 2020; ECHA, 2017; Guo et al., 2019) and some case studies (OECD, 2016a, 2016b, 2018; Nakagawa et al., 2020, 2021) have been reported. However, these approaches can be applied only to specific toxicity end points and substances with a known toxicity and mode of action. Such conditions were previously termed as “local validity” (Patlewicz et al., 2014). 2) The use of biologically similar substances based on their profiles obtained from a large number of bioassays. The United States Environmental Protection Agency’s (US EPA’s) research project, ToxCast and Tox21, provided hundreds of high-throughput screening assays, and several groups employed such biological activity data for toxicological evaluation (Sipes et al., 2013; Berggren et al., 2015; Richard et al., 2021). Although this concept could be applied to substances with little information to elucidate their entire toxicological profiles and find their key mode of action, it is time-consuming and expensive to conduct numerous bioassays for a new candidate substance. By contrast, transcriptome data containing approximately 30,000 gene expression values can be used to estimate perturbated mechanisms through enrichment analysis. Wang et al. (2016) tried to predict drug-induced adverse effects by employing LINCS L1000 data (Subramanian et al., 2017), whereas Iwata et al. (2019) developed a computational method to predict missing values from the LINCS L1000 transcriptomic profiles of various human cell lines and provided new drug therapeutic indications. Genomic data have been considered to be usable in read-across by Health Canada and a research group from the US FDA (Health Canada, 2019; Liu et al., 2019). However, several researchers have shown that *in vitro* gene expression values are not always highly correlated with *in vivo* data (Sutherland et al., 2016; Grinberg et al., 2018; Liu et al., 2018). Thus, interpreting toxicological meaning from the *in vitro*–*in vivo* relationship and *in vitro* to *in vivo* extrapolation (IVIVE) in omics data represents a big challenge for chemical risk assessment. IVIVE was originally researched in toxicokinetics, such as in hepatic clearance and metabolites using hepatocytes (Soars et al., 2007; Umehara and Camenisch, 2012); most recent studies on non-animal testing have focused on predicting plasma concentrations, which is relevant for identification of a margin of exposure in risk assessment (Thomas et al., 2013a; Bell et al., 2018; Li et al., 2021). However, IVIVE should be considered for both toxicokinetics and toxicodynamics. Understanding of the *in vitro* to *in vivo* relationship of bioactivity data is also essential for non-animal testing. As an IVIVE study in omics data, Liu et al. (2020) developed a useful *in silico* strategy to narrow the data gap between *in vitro* and *in vivo* conditions. They modified *in vitro* data using non-generative matrix factorization methods to improve the correlation with *in vivo* data, which overcame the shortcomings of previous large-scale genomic data predictions regarding the *in vitro*–*in vivo* data gap (Liu et al., 2020). Although non-generative matrix factorization enables macroscopic estimation based on a pattern recognition classifying chemical and biological responses, it does not focus on estimation of each gene. As an alternative solution, microscopic estimation of each gene expression was performed based on tensor-train weighted optimization using machine learning (Iwata et al., 2019); however, such comprehensive estimations have not been integrated within an IVIVE study. Therefore, predicting *in vivo* transcriptomic profiles from *in vitro* data for IVIVE might not only enhance the robustness of read-across but could also be utilized in other non-animal testing strategies as weight of evidence, such as in Integrated Approaches to Testing and Assessment (IATA) and new approach methods (NAMs) for safety and drug repositioning research.

In this study, we developed a virtual DNA microarray that quantitatively predicts the *in vivo* gene expression profiles based on the chemical structure and/or *in vitro* transcriptome data. For each gene, elastic-net models, a regression analysis method that has been used in toxicity prediction with visualization of feature importance (e.g., Fujita et al., 2020), were constructed using chemical descriptors and *in vitro* transcriptome data. We named the set of prediction models “regression analysis–based inductive DNA microarray (RAID),” which inductively analyzes the mode of action and the key event in adverse effects with reference to the redundant arrays of inexpensive disks (also represented as RAID), a data storage virtualization technology that combines multiple physical disk drive components with the purpose of data redundancy. As RAID (storage technology) complements data based on information of multiple components, we hope that RAID (our microarray) will complement the relationships between multiple media (*in vivo* gene expression, *in vitro* gene expression, and chemical structure). Our RAID system achieved a quantitative *in vitro* to *in vivo* extrapolation (QIVIVE) by the integration of a structure-based approach (QSAR) with transcriptomic data. Whereas general “Q”IVIVE studies predict dose (or concentration) quantitatively in toxicological or toxicokinetic effects, our “Q”IVIVE predicts *in vivo* gene expression values quantitatively. Finally, the substance similarities were analyzed by principal component analysis (PCA), which proved useful in understanding the features of toxic substances based on their gene expression profile (Watanabe et al., 2012), using RAID (the virtual microarray) data, *in vivo* data, *in vitro* data, and chemical structure data to validate the usefulness of read-across.

## Materials and Methods

### Gene Expression and Chemical Structure Data

No animal experiment was performed in this study. The transcriptome data from DNA microarrays (Affymetrix Rat Genome 230 2.0 chips; Santa Clara, CA, United States) were extracted from the Toxicogenomics Project-Genomics Assisted Toxicity Evaluation system (TG-GATEs). TG-GATEs contains *in vitro* and *in vivo* transcriptome data for rat single- and repeated-dose toxicity tests of 170 compounds (Igarashi et al., 2015). The transcriptome data obtained from the livers of rats treated with high doses for 28 days and primary rat hepatocytes treated with high doses for 24 h were downloaded and preprocessed using MAS5 (Gautier et al., 2004). In this study, chemical substances tested *in vitro* and *in vivo* those fulfilled a maximum sample number (*n* = 2 for *in vitro* and *n* = 3 for *in vivo*) and had no incalculable chemical descriptors (described below) were analyzed. Thus, 115 compounds were examined in this study (Table 1).

**TABLE 1:** List of chemical substances used in the present study and their toxicological classes.

Toxicological class^a^	Name
Toxic	Allyl alcohol (AA), 2-acetamidofluorene (AAF), α-naphthyl isothiocyanate (ANIT), Acetaminophen (APAP), Aspirin (ASA), Benzbromarone (BBr), Bromobenzene (BBZ), Bucetin (BCT), Bendazac (BDZ), Benziodarone (BZD), Carboplatin (CBP), Coumarin (CMA), Chlormezanone (CMN), Chloramphenicol (CMP), Colchicine (COL), Cyclophosphamide monohydrate (CPA), Clomipramine hydrochloride (CPM), Chlorpropamide (CPP), Cyclosporine A (CPA), Diltiazem hydrochloride (DIL), Disopyramide (DIS), Disulfiram (DSF), Dantrolene sodium hemiheptahydrate (DTL), Diazepam (DZP), Ethambutol dihydrochloride (EBU), 17-α-Ethinylestradiol (EE), DL-Ethionine (ET), Fenofibrate (FFB), Flutamide (FT), Gemfibrozil (GFZ), Hexachlorobenzene (HCB), Lomustine (LS), Mexiletine hydrochloride (MEX), Methapyrilene hydrochloride (MP), Methyltestosterone (MTS), Methimazole (MTZ), Nimesulide (NIM), Phenacetin (PCT), Promethazine hydrochloride (PMZ), Propylthiouracil (PTU), Sulfasalazine (SS), Simvastatin (SST), Sulindac (SUL), Thioacetamide (TAA), Terbinafine hydrochloride (TBF), Ticlopidine hydrochloride (TCP), Trimethadione (TMD), Vitamin A (VA), WY-14643 (WY)
Non-toxic	Acarbose (ACA), Acetazolamide (ACZ), Adapin (ADP), Ajmaline (AJM), Amiodarone hydrochloride (AM), Amitriptyline hydrochloride (AMT), Allopurinol (APL), 2-Bromoethylamine hydrobromide (BEA), Caffeine (CAF), Captopril (CAP), Carbamazepine (CBZ), Clofibrate (CFB), Chlorpheniramine maleate (CHL), Cimetidine (CIM), Chlormadinone acetate (CLM), Cephalothin sodium (CLT), Ciprofloxacin hydrochloride (CPX), Chlorpromazine hydrochloride (CPZ), Diclofenac sodium (DFNa), Danazol (DNZ), Erythromycin ethylsuccinate (EME), Enalapril maleate (ENA), Ethanol (ETN), Etoposide (ETP), Famotidine (FAM), Fluphenazine dihydrochloride (FP), Furosemide (FUR), Glibenclamide (GBC), Griseofulvin (GF), Gentamicin sulfate (GMC), Haloperidol (HPL), Hydroxyzine dihydrochloride (HYZ), Ibuprofen (IBU), Imipramine hydrochloride (IMI), Isoniazid (INAH), Iproniazid phosphate (IPA), Ketoconazole (KC), Methyldopa (MDP), Mefenamic acid (MEF), Metformin hydrochloride (MFM), Moxisylyte hydrochloride (MXS), Nitrofurantoin (NFT), Nitrofurazone (NFZ), Nicotinic acid (NIC), Nifedipine (NIF), Omeprazole (OPZ), Papaverine hydrochloride (PAP), Phenobarbital sodium (PB), D-penicillamine (PEN), Perhexiline maleate (PH), Phenylbutazone (PhB), Phenytoin (PHE), Pemoline (PML), Quinidine sulfate (QND), Ranitidine hydrochloride (RAN), Rifampicin (RIF), Sulpiride (SLP), Tannic acid (TAN), Tetracycline hydrochloride (TC), Tiopronin (TIO), Tolbutamide (TLB), Tamoxifen citrate (TMX), Triamterene (TRI), Thioridazine hydrochloride (TRZ), Triazolam (TZM), Sodium valproate (VPA)

a The toxicological classes of chemical substances were referred to in a previous report (Low et al., 2011). The authors classified these substances into histopathological and serum chemistry classes. Substances with hepatotoxic histopathological findings and other histopathological findings with biochemical marker changes in serum chemistry were defined as toxic substances in this study.

For the chemical structure data, the alvaDesc chemical descriptors (Mauri, 2020) were calculated using alvaDesc v1.0 software (Alvascience Srl, Lecco, Italy). AlvaDesc can calculate 3,885 2D-descriptors and 1,420 3D-descriptors. However, only 2D-descriptors were used, excluding those with a high pair correlation (>0.95), constant for all substances, and at least one missing value. Consequently, 854 descriptors were calculated. Each descriptor was normalized using the bestNormalize package (ver. 1.8.0) in R (ver. 4.1.1) (https://cran.r-project.org/). This package estimates the optimal normalizing transformation from the Yeo–Johnson transformation, the Box Cox transformation, the log_10_ transformation, the square root transformation, and the arcsine transformation.

### Construction of the Regression Analysis–Based Inductive DNA Microarray System (Virtual Microarray)

**FIGURE 1:**
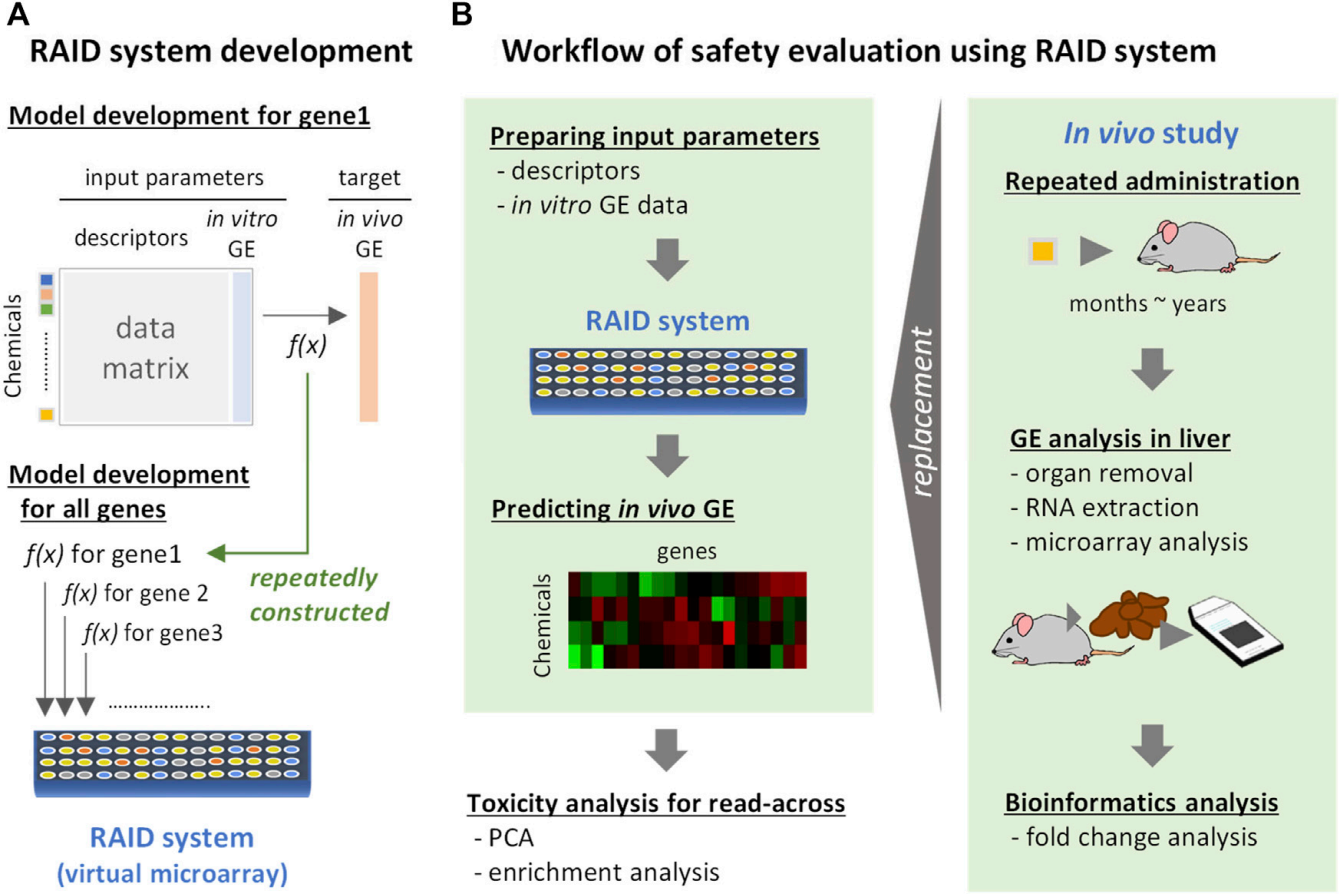
Development and implementation of a virtual microarray (RAID) for read-across. GE: gene expression. f(x): predictive models (formula). **(A)** RAID system development. The predictive model for *in vivo* transcriptome data for each gene was individually constructed by elastic net regression employing chemical descriptors and *in vitro* data. The models constructed were defined as a RAID system (a virtual microarray). **(B)** Workflow of safety evaluation using the RAID system. Chemical descriptors and *in vitro* gene expression data were inputted to the RAID system and *in vivo* gene expression data were outputted. The predicted results were analyzed by PCA and enrichment analysis for read-across. This procedure would replace toxicogenomics analysis in *in vivo* repeated dose study.

To extrapolate *in vitro* transcriptome data to *in vivo* conditions, we developed predictive models for each gene. The predictive models predicting *in vivo* transcriptome data from chemical descriptors and *in vitro* data were developed using the elastic net regression method. The value of each cell in the matrix was the fold change on a base 2 logarithmic scale. The set of those predictive models was named a virtual microarray “RAID” (as mentioned in the *Introduction* section) (Figure 1). To suppress overlearning, the hyperparameters (α and λ) of each model were optimized with a 5-fold cross-validation. We removed the genes that were associated with less than 10 chemical substances inducing differential expression (<1.5-fold change) since it would be difficult to run machine learning scripts on such rare genes. Consequently, RAID was composed of 1,601 prediction models for each gene.

To construct RAID that correctly predicts the bioactivities of chemical substances, the quality of training data sets was extremely important, and differentially expressed genes should be determined strictly considering data noise. Hence, we addressed this issue by data processing (feature engineering) and model justification. First, after calculating the fold change values (sample treated groups/solvent control group), the gene differentiation values with low reliability were adjusted. Briefly, the fold change value increments were changed to half (e.g., 1.5 decreased to 1.25) in the sample with the number of flag A (low reliability) ≥2 out of 3 for *in vivo* and the number of flag A ≥1 out of 2 for *in vitro*, or in the sample with *p*-values ranging between 0.05 and 0.1. The fold change values were changed to one-fourth (e.g., 1.4 decreased to 1.1) in the sample with *p*-value over 0.1 and were treated as 1 (no differentiation) in the sample with flags all A in both *in vivo* and *in vitro*. Second, the weight parameters were used in model building. The weight of samples with ≥1.5-fold change was set to 1.5 and ≥4-fold change was set to 2.

### Interpretation of Biological Meaning of Regression Analysis–Based Inductive DNA Microarray Analysis

Considering the application of RAID to read-across, the gene expression data were visualized by PCA using prcomp function from stats package (ver. 4.1.1), and the probability ellipse frames of toxic and nontoxic substances were drawn using the ggfortify package (ver. 0.4.12) in R to compare *in vivo*, *in vitro*, and chemical descriptor data. The toxic class of chemical substances was determined based on previously reported histopathological and serum chemistry findings (Table 1) (Low et al., 2011). Since PCA did not use the toxicity label for classification, partial least squares discriminant analysis (PLS-DA) using the hepatotoxicity label was also conducted to confirm predictive performance (see Supplementary Material). As a reference data point, the biological meaning of genes that contributed to the PCA plot of *in vivo* data was analyzed using pathway analysis. The loading value of genes in the PCA was defined as length of loadings calculated using the Pythagorean theorem:

$$length=\;\sqrt{{\left(loading\;of\;PC1\right)}^{2}+\;{\left(loading\;of\;PC2\right)}^{2}},$$

and genes with the top 30 loading values in the first and fourth quadrant were analyzed.

To analyze the biological consistency with *in vivo* data, commonality of principal component–related genes (top and bottom 30 rotations in each PC1 and PC2 of PCA) were visualized using the VennDiagram package (ver. 1.6.20) in R, and enrichment analyses of each categorized gene were conducted using Gene Ontology—biological process and Reactome pathway by Metascape (Zhou et al., 2019). Four categorized genes related to *in vivo* data (*in vivo* only, *in vivo* and RAID, *in vivo* and *in vitro*, and all three data) were analyzed to characterize which biological process could be covered by RAID and *in vitro* data. Furthermore, to characterize genes whose predictive models in RAID used *in vitro* data, enrichment analysis of the top 20 genes with the highest importance (contribution) for *in vitro* data in the model was conducted. In the analysis, the Affymetrix probe ID was converted to gene symbol using the biomaRt package (ver. 2.50.2) in R.

### Quantitative *In Vitro* to *In Vivo* Extrapolation Effects in Regression Analysis–Based Inductive DNA Microarray System

For performance evaluation against the quantitative IVIVE, root-mean-square errors (RMSEs) of RAID predicted values to *in vivo* data were calculated and compared to those of *in vitro* data. To exclude the differences in gene expression value distribution of each data source, the fold change values were normalized before the RMSEs were calculated. The RMSEs were calculated both for all genes and genes for which *in vitro* data had importance in the model.

### Read-Across Application Using External Data

To validate the usefulness of RAID for functional read-across–based analysis of both predicted gene expression profiles and chemical structures, substances that did not contain training data sets for model building (Table 1) were further explored using Ingenuity Pathway Analysis (IPA) (QIAGEN Inc., https://www.qiagenbioinformatics.com/products/ingenuitypathway-analysis). Specifically, substances that may promote the expression of genes (having a known relationship with the gene) that were identified by the PCA and pathway analysis of *in vivo* data (see the *Interpretation of Biological Meaning of Regression Analysis–Based Inductive DNA Microarray Analysis* section) were explored using IPA. Chemical descriptors of each substance were analyzed using the alvaDesc v1.0 software (Alvascience Srl, Lecco, Italy), and the gene expression profiles were fulfilled using median values of training data sets. Finally, RAID analyses using constructed predictive models for those substances and reanalyzed PCA data were used to evaluate similarities based on the predicted biological responses.

## Results

### Biological Analysis of Regression Analysis–Based Inductive DNA Microarray Compared to That of *In Vivo* and *In Vitro* Microarray Data

**FIGURE 2:**
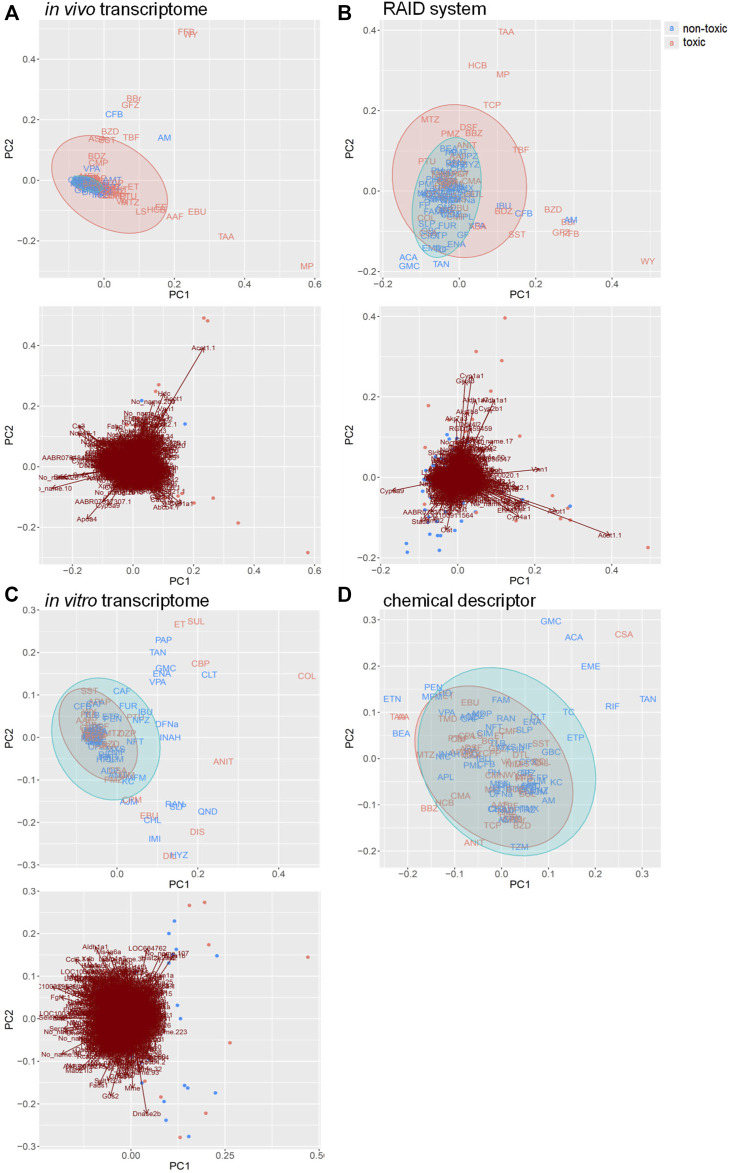
PCA score plots for chemical substances and the gene loading in the transcriptome data of **(A)** *in vivo*, **(B)** virtual microarray (RAID), and **(C)** *in vitro* data. PCA score plot with **(D)** chemical descriptor data. Uppercase letters in PCA score plots: abbreviations of chemical substances are described in Table 1. Blue: nontoxic substances. Red: hepatotoxic substances. Gene symbols are presented on the arrowhead (loading).

RAID (predicted transcriptome) data were visualized using PCA (Figure 2). From a higher perspective, two directions mainly composed of toxic substances were identified, and many toxic substances were separated from non-toxic substances *via* RAID and *in vivo* data, whereas they could not be separated based on *in vitro* and chemical descriptor data. Moreover, two common toxic substances groups [e.g., first group (TAA, MP, and HCB) and second group (WY, FFB, BBr, and GFZ) placed in the first and fourth quadrants) were distanced from non-toxic substances along PC1 and PC2 in both RAID and *in vivo* data, nonetheless the PC1 and PC2 were replaced. The loading plot showed that *Cyp1a1 (cytochrome P450, family 1, subfamily A, polypeptide 1)*, *Gpx2 (glutathione peroxidase 2)*, and *Gsta3 (glutathione S-transferase A3)* gene expressions were commonly observed in RAID and in *in vivo* data and enabled the discrimination of TAA, MP, and HCB. Furthermore, *Acot1 (acyl-CoA thioesterase 1)*, *Vnn1 (vanin 1),* and *Cyp4a11 (cytochrome P450, family 4, subfamily A, polypeptide 11)* contributed to discriminating WY, FFB, BBr, and GFZ.

**FIGURE 3:**
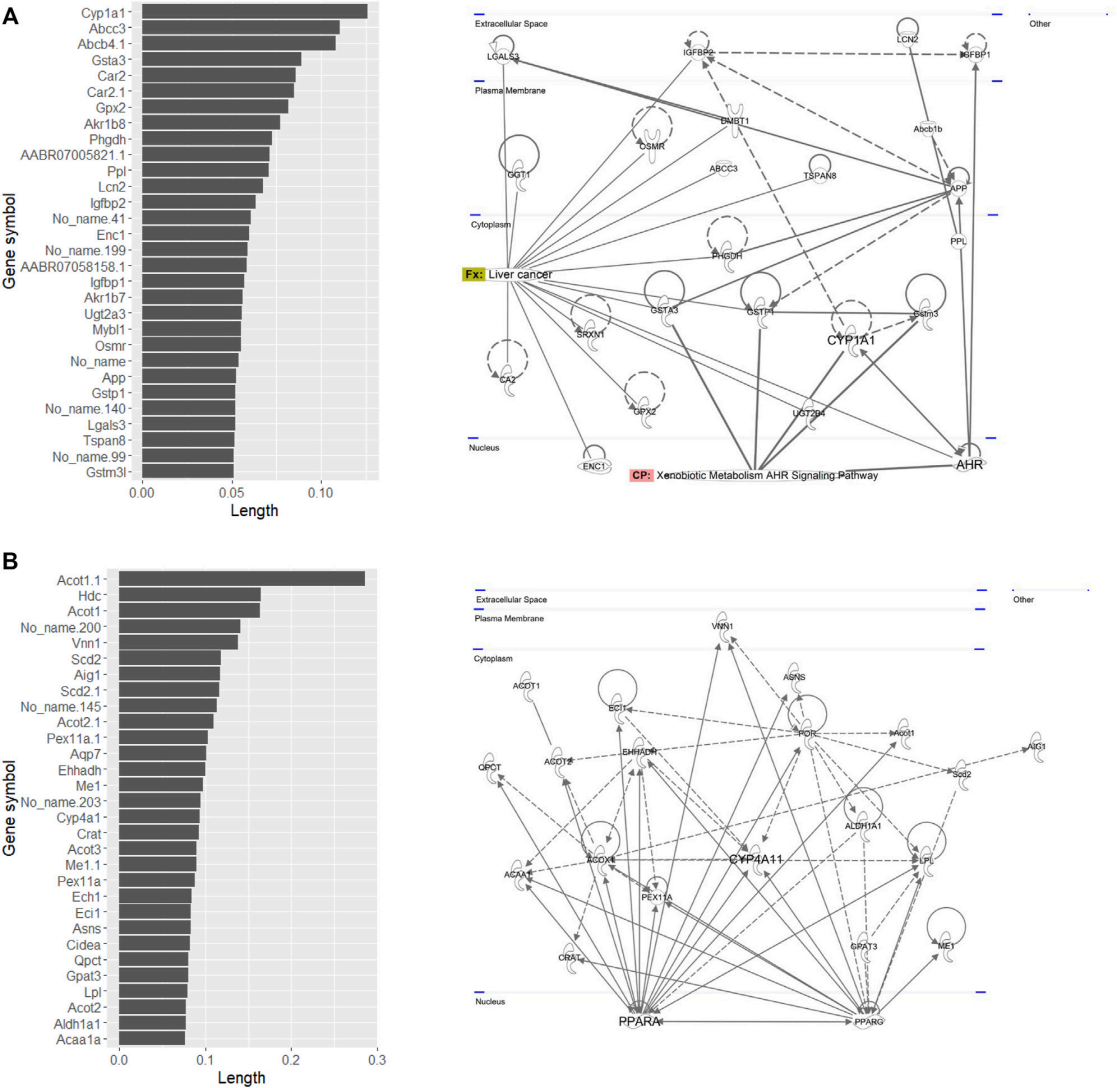
List of genes that have high loading values in the **(A)** fourth quadrant and **(B)** first quadrant in the PCA plot of *in vivo* data, where the first group (TAA, MP, and HCB) and the second group (WY, FFB, BBr, and GFZ) plotted, and their pathway map. The loading value was defined as the loading length in the first or fourth quadrant calculated using the Pythagorean theorem. The pathway map was drawn by upstream regulator analysis using IPA.

**FIGURE 4:**
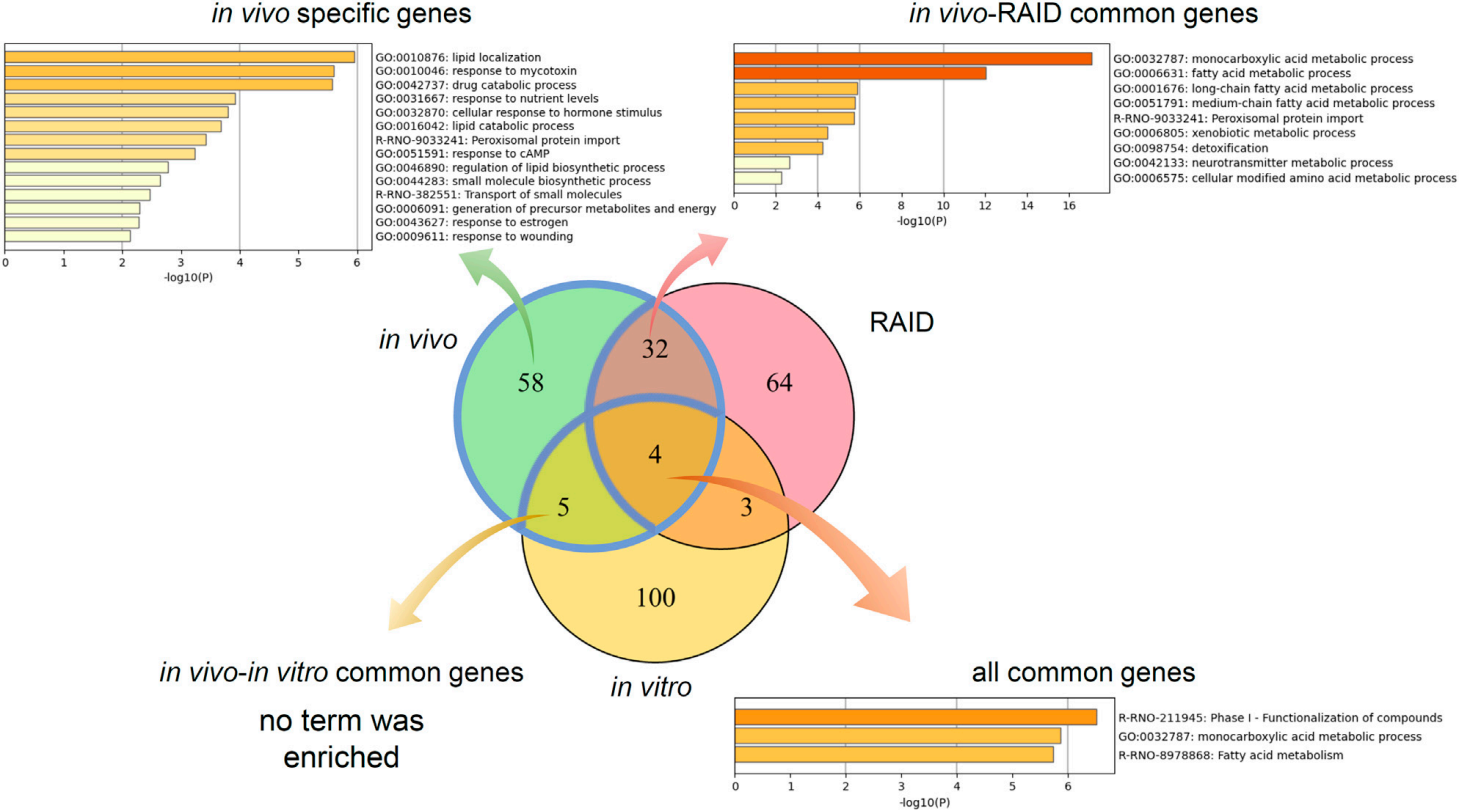
Commonalities of principal component–related genes and their biological functions analyzed by gene ontology and pathway analyses. Venn diagram of genes related to the first and second principal components of *in vivo*, a virtual microarray (RAID), and *in vitro* data.

Pathway analysis indicated that the first group–related genes would be associated with a xenobiotic response, such as *Cyp1a* induction *via* aryl hydrocarbon receptor (AHR) and carcinogenesis (Figure 3A), and the second group–related genes would be associated with peroxisome proliferative activity characterized by *Cyp4a* induction *via* peroxisome proliferator–activated receptor-alpha (PPARa) activation (Figure 3B). To clarify the biological functions that RAID covers, the commonalities between the related genes and principal components were explored (Figure 4 and Table 2). As expected from Figure 2, RAID shared more genes (36; Table 2) with the *in vivo* data than with the *in vitro* data (9). Enrichment analysis revealed that the biological processes related to metabolism and detoxification and pathways associated with peroxisomal protein transport were enriched in both *in vivo* and RAID data, indicating that RAID could cover these functions, and ultimately indicate key functions through pathway analysis (Figure 3). Conversely, although several metabolic processes were enriched within the *in vitro* data, those biological functions were covered by RAID as well (Figure 4). These results suggest that RAID data allow the detection of more *in vivo* key toxic events than *in vitro* transcriptome data.

**TABLE 2:** Principal components relating common genes in a virtual microarray (RAID) and *in vivo* data.

Probe ID	Symbol	Description
1398250_at	Acot1	Acyl-CoA thioesterase 1
1370269_at	Cyp1a1	Cytochrome P450, family 1, subfamily a, polypeptide 1
1387022_at	Aldh1a1	Aldehyde dehydrogenase 1, family member A1
1368934_at	Cyp4a1	Cytochrome P450, family 4, subfamily a, polypeptide 1
1388211_s_at	Acot1	Acyl-CoA thioesterase 1
1374070_at	Gpx2	Glutathione peroxidase 2
1367811_at	Phgdh	Phosphoglycerate dehydrogenase
1389253_at	Vnn1	Vanin 1
1388210_at	Acot2	Acyl-CoA thioesterase 2
1371089_at	Gsta3	Glutathione S-transferase alpha 3
1370491_a_at	Hdc	Histidine decarboxylase
1379275_at	Snx10	Sorting nexin 10
1370902_at	Akr1b8	Aldo-keto reductase, family 1, member B8
1367733_at	Car2	Carbonic anhydrase
1386889_at	Scd2	Stearoyl-Coenzyme A desaturase 2
1386901_at	LOC103690020	Platelet glycoprotein 4-like
1391187_at	Ppl	Periplakin
1384225_at	Dab1	DAB adaptor protein 1
1384274_at	AABR07037307	similar to Spindlin-like protein 2
1395403_at	Stac3	SH3 and cysteine-rich domain 3
1375845_at	Aig1	Androgen induced 1
1368283_at	Ehhadh	Enoyl-CoA hydratase and 3-hydroxyacyl CoA dehydrogenase
1387740_at	Pex11a	Peroxisomal biogenesis factor 11 alpha
1370067_at	Me1	Malic enzyme 1
1370870_at	Me1	Malic enzyme 1
1371886_at	Crat	Carnitine O-acetyltransferase
1379361_at	Pex11a	Peroxisomal biogenesis factor 11 alpha
1386885_at	Ech1	Enoyl-CoA hydratase 1
1367659_s_at	Eci1	Enoyl-CoA delta isomerase 1
1378169_at	Acot3	Acyl-CoA thioesterase 3
1374475_at	Abhd1	Abhydrolase domain containing 1
1387783_a_at	Acaa1a	Acetyl-Coenzyme A acyltransferase 1A
1390591_at	Slc17a3	Solute carrier, family 17, member 3
1368607_at	Cyp4a8	Cytochrome P450, family 4, subfamily a, polypeptide 8
1370698_at	Ugt2b10	UDP-glucuronosyltransferase, family 2, member B10
1370387_at	Cyp3a9	Cytochrome P450, family 3, subfamily a, polypeptide 9

For performance confirmation of discriminative analysis for hepatotoxicity, PLS-DA using RAID data allowed us to separate toxic chemicals with high accuracy (Supplementary Table S1). The accuracy using RAID data was better than that without RAID, when calibration and test data set were prepared.

### Importance of *In Vitro* Data in the Regression Analysis–Based Inductive DNA Microarray System

**FIGURE 5:**
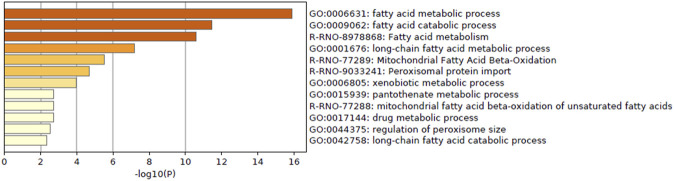
Enrichment analysis of *in vitro–in vivo* extrapolation (IVIVE)–related genes identified in a virtual microarray (RAID) system. Top 20 most important (contribution) genes from the predictive models were analyzed.

Enrichment analysis of genes whose predictive model used highly relevant *in vitro* data (top 20 genes for which *in vitro* data had high importance in all predictive models; Table 3) indicated that *in vitro* data contributed to estimating the gene expression values associated with metabolic processes of fatty acids, xenobiotics, and drugs and peroxisome proliferative activity (pathway on peroxisome protein import and biological processes associated with the regulation of peroxisome size; Figure 5).

**TABLE 3:** List of top 20 genes with high importance *in vitro* data in the predictive models in RAID.

Probe ID	Symbol	Description	Importance of *in vitro* data
1398250_at	Acot1	Acyl-CoA thioesterase 1	0.550
1368934_at	Cyp4a1	Cytochrome P450, family 4, subfamily a, polypeptide 1	0.412
1367659_s_at	Eci1	Enoyl-CoA delta isomerase 1	0.360
1368283_at	Ehhadh	Enoyl-CoA hydratase and 3-hydroxyacyl CoA dehydrogenase	0.348
1387740_at	Pex11a	Peroxisomal biogenesis factor 11 alpha	0.314
1370269_at	Cyp1a1	Cytochrome P450, family 1, subfamily a, polypeptide 1	0.284
1386885_at	Ech1	Enoyl-CoA hydratase 1	0.252
1389253_at	Vnn1	Vanin 1	0.244
1387783_a_at	Acaa1a	Acetyl-Coenzyme A acyltransferase 1A	0.238
1371076_at	Cyp2b1	Cytochrome P450, family 2, subfamily a, polypeptide 1	0.220
1375845_at	Aig1	Androgen induced 1	0.166
1388211_s_at	Acot1	Acyl-CoA thioesterase 1	0.127
1379361_at	Pex11a	Peroxisomal biogenesis factor 11 alpha	0.125
1386901_at	LOC103690020	Platelet glycoprotein 4-like	0.115
1370397_at	Cyp4a3	Cytochrome P450, family 4, subfamily a, polypeptide 3	0.114
1386880_at	Acaa2	Acetyl-CoA acyltransferase 2	0.096
1384244_at	Hsdl2	Hydroxysteroid dehydrogenase like 2	0.074
1370698_at	Ugt2b10	UDP glucuronosyltransferase, family 2, member B10	0.073
1397468_at	Hsdl2	Hydroxysteroid dehydrogenase like 2	0.071
1367777_at	Decr1	2,4-dienoyl-CoA reductase 1	0.070

### Quantitative *In Vitro* to *In Vivo* Extrapolation Performance in the Regression Analysis–Based Inductive DNA Microarray System

**FIGURE 6:**
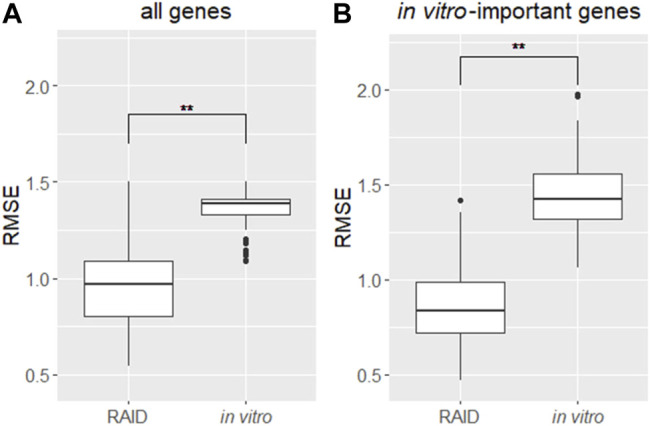
Distribution of RMSEs of a virtual microarray (RAID) and *in vitro* data of **(A)** all genes and **(B)** *in vitro* genes having importance (contribution) in predictive models. **p < 0.01 (Welch’s t-test).

To evaluate RAID performance in terms of gene expression values, the RMSEs were calculated for all genes and the genes for which *in vitro* data had importance in predictive models. Considering RAID would be used in read-across, we compared the RMSEs of RAID data with those of *in vitro* data, from conventional non-animal test approaches (Figure 6). The RMSEs were lower in RAID, indicating a better performance than what could be obtained using *in vitro* data.

### Validation of Prediction Models Using External Data

In PCA with *in vivo* and RAID data, as well as the pathway analysis of PC-related genes (Figures 2, 3), the genes related to peroxisome proliferative activity and xenobiotic metabolism activity possibly leading to liver cancer, which were respectively characterized by *Cyp1a* induction *via* AHR and *Cyp4* induction *via* PPARa, were identified as key features. Thus, potential *Cyp1a* and *Cyp4a* inducers were explored using the knowledge-based approach using the IPA software. Moreover, using the top 30 genes identified using PCA (described in the *Interpretation of Biological Meaning of Regression Analysis–Based Inductive DNA Microarray Analysis* section), upstream regulator analysis focusing on chemical substances was performed, and 20 chemicals were identified. Finally, a total of 21 chemicals (potential *Cyp1a* inducers: 10 chemicals; potential *Cyp4a* inducers: 11 chemicals) were selected as candidates for external validation and were subjected to RAID analyses (Table 4). Substances already present in the TG-GATE (training sets) or had uncalculated chemical descriptors data were excluded.

**TABLE 4:** List of chemical substances used for external validation of the RAID system.

Name	CAS no.	Name in PCA plot
Potential Cyp1a inducers		
2,3,4,7,8-Pentachlorodibenzofuran	57117-31-4	Pentachlorodibenzofuran
3,4,5,3′,4′-Pentachlorobiphenyl	57465-28-8	Pentachlorobiphenyl
3-Methylcholanthrene	56-49-5	Methylcholanthrene
9,10-Dimethyl-1,2-benzanthracene	57-97-6	Dimethylbenzanthracene
Benzo(a)pyrene	50-32-8	Benzo(a)pyrene
Dexamethasone	8054-59-9	Dexamethasone
Genistein	446-72-0	Genistein
2,2′,4,4′-Tetrachlorobiphenyl	1336-36-3	Tetrachlorobiphenyl
Quercetin	117-39-5	Quercetin
Resveratrol	501-36-0	Resveratrol
Thiabendazole	148-79-8	Thiabendazole
Potential Cyp4a inducers		
Streptozotocin	18883-66-4	Streptozotocin
2-Ethylhexanol	104-76-7	Ethylhexanol
Di(2-ethylhexyl) phthalate	117-81-7	Di(2-ethylhexyl)_phthalate
Clofenapate	21340-68-1	Clofenapate
Clofibric acid	882-09-7	Clofibric_acid
Ciprofibrate	52214-84-3	Ciprofibrate
Nafenopin	3711-19-5	Nafenopin
TO-901317	293754-55-9	TO-901317
Acetaminophen	719293-04-6	Acetaminophen
Diltiazem	33286-22-5	Diltiazem

**FIGURE 7:**
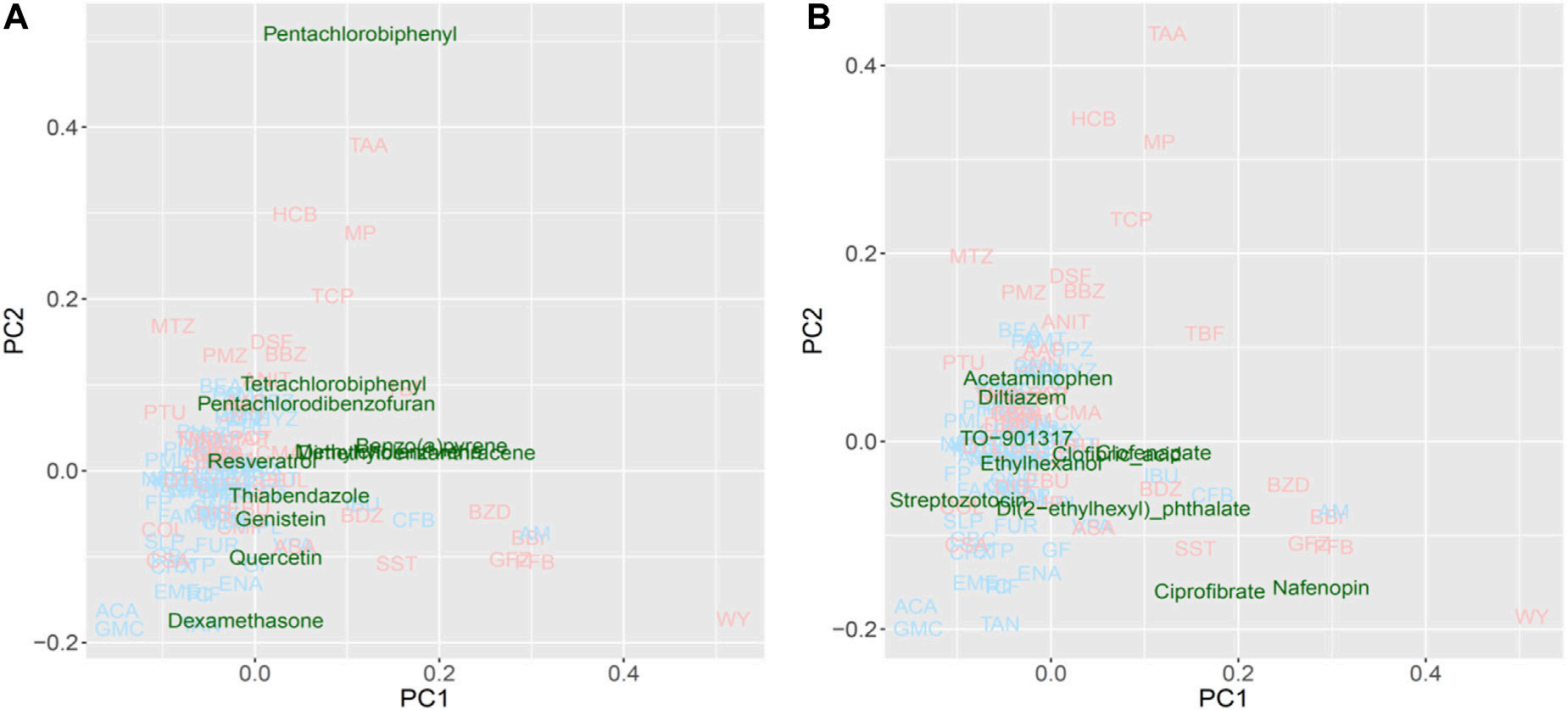
Read-across using PCA plot of external data predicted by a virtual microarray (RAID). **(A)** *Cyp1a* and **(B)** *Cyp4a* inducing chemical substances were analyzed for validation.

For the PCA, approximately half of the substances were plotted with positive PC scores, which was consistent with the direction expected from the training data set for both potential *Cyp1a* and *Cyp4a* inducers (Figure 7). Lastly, pentachlorobiphenyl, polychlorinated biphenyls, and pentachlorodibenzofuran were isolated as *Cyp1a* inducers, whereas nafenopin, ciprofibrate, and di(2-ethylhexyl)phthalate were isolated as *Cyp4a* inducers.

## Discussion

The transcriptome data signatures derived from the RAID (the virtual microarray) system were in good agreement with those of *in vivo* data, and the technology provided an understanding of the features of hepatotoxic substances based on the toxicological mechanism interpretation. The mechanism of action of the two characteristic toxic substances separated using PCA analysis was shown to be achieved through *Cyp1a* induction *via* AHR and *Cyp4a* induction *via* PPARa (pathway and gene ontology analysis). The AHR-induced drugs raise safety concerns during developmental periods (Qin et al., 2019), and PPARa-induced drug toxicity requires species differentiation considerations (Ito et al., 2006). Therefore, predicting the involvement of these nuclear receptors and induction of metabolic enzymes is critical for understanding the molecular initiating events and the key events associated with adverse outcome pathway. RAID enables the prediction of gene expression levels, thus exhibiting properties required for the next-generation risk assessment methods.

The first substance group (TAA, MP, and HCB), representing toxic substances commonly differentiated from non-toxic substances using PCA on *in vivo* and RAID data, has been reported to have carcinogenicity with metabolic activation (Uehara et al., 2008; Hajovsky et al., 2012; US HSS, 2015). Furthermore, these substances have been shown to activate xenobiotic-related receptors, such as AHR inducing *Cyp1a* (Ushel et al., 2002; Yamashita et al., 2014; Clara et al., 2015). Moreover, *in vivo* transcriptome data in this study showed that TAA, MP, and HCB induce *Cyp1a* activation. AHR is known for mediating the toxicity and tumor promoting properties despite the mechanism through which AHR activates carcinogenesis needing to be elucidated (Safe et al., 2013; Murray et al., 2014).

The second substance group (WY, FFB, BBr, and GFZ) includes fibrates which are recognized as PPARa agonists (Schoonjans et al., 1996), implying that induction of *Cyp4a via* PPARa and perturbation of lipid-related genes are involved as a series of key events. Although another fibrate included in training data—clofibrate (CFB)—was classified as a non-toxic substance according to no serum chemistry findings from a previous study, CFB was shown to act as a PPARa agonist inducing peroxisomal proliferation on hepatocytes (Low et al., 2011) and was plotted around the second group in the PCA. Sustained activation of PPARa signaling and induction of enzymes, such as CYP4A, to increased fatty acid oxidation contributes to sustained oxidative stress in the liver. These changes lead to liver cell damage as hypertrophy and proliferation which contribute to the development of hepatocellular carcinoma (Parimal et al., 2013).

From the perspective of capturing individual gene responses, RAID could detect gene expressions related to major drug metabolism responses in *in vivo* more broadly (more common principal component–related gene number; Figure 4) and quantitatively (lower RMSE values; Figure 6) than *in vitro*. The 36 genes that were commonly related to the principal components of *in vivo* and RAID data contained genes that were known to be involved in drug metabolism and hepatotoxicity. In addition to the genes described above (*Cyp1a* and *Cyp4a*), *Acot1* acts as an auxiliary enzyme in the oxidation process of various lipids in peroxisomes (Hunt et al., 2012). Furthermore, *Vnn1* is expressed by the centrilobular hepatocytes and is involved in lipid and xenobiotic metabolism (Bartucci et al., 2019), whereas *Pex11a* (*peroxisomal biogenesis factor 11 alpha*) is involved in peroxisome maintenance and proliferation associated with dyslipidemia (Chen et al., 2018). All of these genes are known as PPARa target genes (Rakhshandehroo et al., 2010; Lake et al., 2016). Thus, these features indicate that RAID can predict possible toxicity by taking into account a broader range of mechanisms than the range of *in vitro* data. Indeed, the *in vivo* changes detected using the *in vitro* data were limited (Figure 4), and the PCA showed that most of the differentially expressed genes were associated with irrelevant nonphysiological conditions. Thus, the IVIVE effect combining the QSAR technique and *in vitro* data would allow for more precise predictions through de-noising these types of *in vitro* specific biological responses.

*In vitro* data contribute to accurate gene expression predictions that could not be achieved with QSAR alone (Figure 2D). *In vitro* data contributed to the prediction of the mechanism shown in Figure 5. The biological mechanisms related to metabolic processes were consistent with the key mechanisms of characteristic hepatotoxic substances described above, which indicates that *in vitro* data contributes to the precise predictions obtained using RAID. In addition, whether *in vitro* responses were observed in the suggested mode of action predicted by the RAID system or not is an important point in terms of weight of evidence. This study provides valuable evidence supporting that transcriptome data should be considered in light of previous reports indicating that *in vitro* data does not necessarily reflect *in vivo* conditions (Tamura et al., 2006; Sutherland et al., 2016). Simultaneously, *in vitro* studies focusing on a specific mechanism should consider the external validity of their findings and whether the findings reflect *in vivo* situations.

Evaluating the read-across performance using external substances, such as 3,4,5,3′,4′-pentachlorobiphenyl, 2,2′,4,4′-tetrachlorobiphenyl (a type of polychlorinated biphenyl) and pentachlorodibenzofuran (dioxin-like compounds) (Figure 7A), which are known as IARC group 1 carcinogens and *Cyp1a1* inducers (EPA,U S, 1996; Walker et al., 2005; National; Toxicology Program, 2006); these were separated as toxic substances. Additionally, benzo(a)pyrene, 3-methylcholanthrene, and 9,10-dimethyl-1,2-benzanthracene plotted apart from the origin of coordinates (PC1 = 0 and PC2 = 0), and are polycyclic aromatic hydrocarbons inducing *Cyp1a1* (Moorthy et al., 2007; Pushparajah et al., 2008). Non-carcinogenic chemical substances, such as food components or preservatives, were positioned near the origin, second quadrant or third quadrant, indicating low risk. Furthermore, substances interacting with *Cyp4a* (Figure 7B), such as ciprofibrate, nafenopin, clofenapate, clofibric acid, and di(2-ethylhexyl) phthalate, which plotted in the area of the 2nd substance group (PC1 > 0), are also known as PPARa agonists (Bocos et al., 1995; Roberts et al., 2002; Yadetie et al., 2003; Currie et al., 2005; Pyper et al., 2010). Chemicals that were not characterized by the PC1 component (PC1 < 0) are not hyperlipidemia drugs. These results suggest that the RAID system effectively classifies substances based on their mode of action as well as the strength of their toxicity, and ultimately contributes to precise read-across. Thus, the RAID system provides a new method for read-across in line with IATA that should be called “a virtual functional read-across”. Here, we showed that substances without high structural similarities might have similar toxicological properties, and our new approach interpreted the shared mechanism of action. This means that RAID considers the qualitative and quantitative similarities of biological responses, which was one of the major issues of QSAR-based read-across. The structural similarities of TAA, MP, and HCB observed using correlation coefficients of the chemical descriptor used for the predictive model, and the maximum common substructure (MCS) similarities with the Tanimoto coefficient, were less than 0.5; however, the homology of RAID and *in vivo* data was as high as a 0.8 Pearson’s correlation coefficient. Furthermore, achieving such an accurate read-across without using *in vitro* data will provide a new perspective on the structural information-based predictions.

PCA analysis was used to understand the features of substances to predict the modes of action and identify biologically similar substances for read-across in this study. The examples of applications of RAID for read-across described above were compared to other methods (Table 5). The RAID system could enhance read-across reliability by estimating toxicity including modes of action, while this was difficult by other methods (e.g., QSAR or read-across using chemical structure data) (Supplementary Figure S1). On the other hand, focusing on certain specific toxicities, discriminant analysis, classifier model, or biomarker analysis might improve the separation of toxic substances. Indeed, as shown in Supplementary Table S1, when the RAID system was applied to discriminating hepatotoxicity, PLS-DA using RAID data showed a good predictive performance, indicating usefulness for the specified toxicity prediction. Thus, the use of RAID data instead of experimental transcriptome data would achieve previously reported biomarker-based classification without using animals. For example, Liu et al. (2017) indicated that certain genes were associated with hepatocellular hypertrophy and hepatocarcinogenesis, as well as markers such as *Cyp1a1*, *Acot1*, *Stac3 (SH3 and cysteine rich domain 3)*, and *Hdc (histidine decarboxylase),* which were correctly evaluated in the present study to characterize hepatotoxic substances. Similarly, the constructed RAID system could be applied to previous studies to predict carcinogenicity or estimate transcriptional benchmark dose by toxicogenomics analysis of short term *in vivo* studies (Ellinger-ziegelbauer et al., 2008; Thomas et al., 2013b; Matsumoto et al., 2014; Kawamoto et al., 2017).

**TABLE 5:** The relationships between the pros and cons of RAID and other methods for read-across.

Examples of chemical substances in the present study	QSAR	Read-across using PCA of chemical structure data	Read-across using PCA of RAID data
Internal data A: TAA B: FFB External data C: 3,4,5,3′,4′-pentachlorobiphenyl D: Nafenopin	Pros. Toxicity may be identified.	Pros. Chemical structure similarity can be calculated easily.	Pros. The toxicity and modes of action of *in vivo* can be estimated from the PCA plot. Animal testing data of similar substances can be utilized for the assessment. A: HCB and MP were similar substances, and it was estimated that TAA could make Cyp1a induction *via* AHR, and substances plotted in the first quadrant would have similar possibilities. B: WY, BBr, and GFZ were similar substances, and it was estimated that FFB could make Cyp4a induction *via* PPARa activation, and substances plotted in the first quadrant would have similar possibilities. C: Its toxicological response could be similar to “TAA, MP, and HCB,” indicating that it could induce Cyp1a. Its carcinogenic potential should be confirmed using further additional testing. D: Its toxicological response could be similar to “WY, FFB, BBr, and GFZ,” indicating that it could induce Cyp4a and also affect the expression of PPARa-related genes.
	Cons. Mechanisms cannot be fully estimated because of the lack of biological activity data. Toxicity in organs and individuals cannot be characterized. Biologically similar substances cannot be identified.	Cons. Estimation of the toxicity and modes of action from the PCA plot is complicated because toxic substances cannot be separated well from non-toxic substances. A, C: Estimation of the toxicity and modes of action was difficult since similar substances were both toxic and non-toxic. B, D: Specific similar substances were not identified since they were surrounded by many substances.	Cons. The reliability of the estimated modes of action would depend on the accuracy of the RAID system.

One important issue that should be considered in toxicological evaluation using the RAID system is consideration of species differences. The RAID system provides mechanistic insights on repeated-dose toxicity in animal models; however, since some species differences have been observed, the suggested mode of action and the corresponding molecules need to be confirmed by toxicologists. The interspecies extrapolations, such as rat-to-human extrapolations, could be achieved by further experiments to construct new RAID systems with these different species’ transcriptome data. In addition, RAID data of substances that were separated as toxic substances in PCA (e.g., TAA, MP, HCB, WY, FFB, BBr, and GFZ) showed high similarity to *in vivo* data (Supplementary Table S2). Since regression analysis requires certain levels of standard deviation of training data, the RAID accuracy for substances may be related to the number of substances with similar modes of action. Thus, database expansion for several substance groups with minor modes of action would contribute to further improving the accuracy and applicability domain. In addition, evaluation of RAID usefulness for various toxicities is required.

The present approach integrates QSAR and IVIVE and will contribute to other areas of research, such as drug repositioning, which has recently attracted attention toward pharmaceuticals that are available on the market and might be repurposed for new diseases (Jourdan et al., 2020). However, the previously proposed methodologies (Iwata et al., 2018; Lippmann et al., 2018; Zhu et al., 2020; He et al., 2021) have room for improving the IVIVE aspect of *in vivo* predictions. Thus, our system provides an alternative to screening candidate drugs and exploring new biologically similar drugs at a low cost.

In conclusion, we developed a virtual DNA microarray system that quantitatively predicts *in vivo* gene expression profiles based on the chemical structure and/or *in vitro* transcriptome data. Estimated transcriptomes are considered scientifically relevant from PCA data interpretation as well as pathway and GO analysis. Based on its external validation, our system works as an alternative test for repeated dose toxicity tests with toxicogenomics analysis enabling IVIVE and mechanism estimation. Although our technology might have limited applicability domain due to the small data size of chemical substances and their characteristics (using hepatotoxic substances), the concept of the virtual microarray analysis contributes to the 3Rs (reduction, refinement, and replacement) and might benefit much future animal testing.

## Data Availability

Publicly available data sets were analyzed in this study. This data can be found at https://dbarchive.biosciencedbc.jp/en/open-tggates/download.html.
