# Supplementary material for: RAID: Regression Analysis–Based Inductive DNA Microarray for Precise Read-Across
Source: Front Pharmacol. 2022 Jul 22;13:879907. doi: 10.3389/fphar.2022.879907 (PMC9354856; doi:10.3389/fphar.2022.879907)
Supplement: Supplementary file 1 [file DataSheet1.docx]

Supplementary Material

**RAID: Regression Analysis-based Inductive DNA Microarray for Precise Read-Across**

**Yuto Amano^1^, Masayuki Yamane^1^, Hiroshi Honda^1^**

^1^R&D Safety Science Research, Kao Corporation, 2606 Akabane, Ichikai–Machi, Haga–Gun, Tochigi, Japan

**Supplementary Table S1.** Accuracy of the PLS-DA for hepatotoxic substances. Accuracies using PLS component 1, 2, and 3 (Comp1, 2, 3) were calculated A) when all data was used and B) when calibration and test dataset were used in PLS-DA for RAID data and chemical descriptor and *in vitro* combined data (Chemical descriptor (CD) + *in vitro* gene expression).

| A) all data |  |  |  |
| --- | --- | --- | --- |
|  | Comp1 | Comp2 | Comp3 |
| RAID data | 0.696 | 0.739 | 0.843 |
| CD + *in vitro* gene expression | 0.678 | 0.843 | 0.904 |

| B) Predict data set |  |  |  |
| --- | --- | --- | --- |
|  | Comp1 | Comp2 | Comp3 |
| RAID data | 0.667 | 0.667 | 0.754 |
| CD + *in vitro* gene expression | 0.614 | 0.561 | 0.614 |

In this study, principal component analysis (PCA) was used to understand the features of substances to predict the modes of action and identify biologically similar compounds for read-across. However, since PCA does not use toxicity label for classification, partial least squares discriminant analysis (PLS-DA) using hepatotoxicity label was conducted. PLS-DA was applied to RAID data or chemical descriptor and *in vitro* combined data (without RAID system) by plsda function from mdatools package (ver. 0.12.0) in R (ver. 4.1.1) (<https://cran.r-project.org/>). The toxic class of chemical substances was the same as in the main text (Table 1). The number of components included to the model was set to three. All chemical data were divided into two equal parts for calibration and test dataset, and accuracies using PLS component 1, 2, and 3 were compared between all data and these datasets.

The accuracies of PLS-DA using RAID data were less than that using CD and *in vitro* gene expression data when all data was used, whereas the relationship was reversed when the calibration and test datasets were used (Supplementary Table S1). Thus, although further model optimization and predicted data analysis are needed to improve predictive performance and make detailed interpretations, RAID data might be appropriate to predict new external substances for safety assessment.

**Supplementary Table S2.** The similarities between RAID and *in vivo* data of TAA, MP, HCB, WY, FFB, BBr, and GFZ and their order within the substances used in this study. The similarities were shown as Pearson’s correlation coefficients of RAID and *in vivo* data.

| Order of similarity between RAID and *in vivo* data | Name | Pearson’s correlation coefficient between RAID and *in vivo* data |
| --- | --- | --- |
| 1 | HCB | 0.85 |
| 3 | GFZ | 0.82 |
| 5 | BBr | 0.81 |
| 6 | TAA | 0.81 |
| 14 | WY | 0.76 |
| 15 | FFB | 0.75 |
| 19 | MP | 0.72 |

A)

B)

**Supplementary Figure S1.** PCA score plots of the chemical descriptor and RAID data of chemical substance. Red and pale pink: toxic substance. Sky blue: non-toxic substance. Green: external substance. Red and green substances were described in Table 5. A) TAA, HCB, MP, and 3,4,5,3',4'-pentachlorobiphenyl, and B) WY, FFB, BBr, GFZ, and Nafenopin were identified as similar in RAID but not in chemical descriptor.
